# Knowledge and attitudes of physicians toward research ethics and scientific misconduct in Lebanon

**DOI:** 10.1186/s12910-020-00475-5

**Published:** 2020-05-14

**Authors:** Bilal Azakir, Hassan Mobarak, Sami Al Najjar, Azza Abou El Naga, Najlaa Mashaal

**Affiliations:** Faculty of Medicine, Beirut Arab University, Beirut, Lebanon

**Keywords:** Knowledge, Attitude, Physicians, Research ethics, Misconduct, Lebanon

## Abstract

**Background:**

Despite the implementation of codes and declarations of medical research ethics, unethical behavior is still reported among researchers. Most of the medical faculties have included topics related to medical research ethics and developed ethical committees; yet, in some cases, unethical behaviors are still observed, and many obstacles are still conferring to applying these guidelines.

**Methods:**

This cross-sectional questionnaire-based study was conducted by interviewing randomly selected 331 Lebanese physicians across Lebanon, to assess their awareness, knowledge and attitudes on practice regarding international and national research ethics guidelines (Lebanese decrees/Laws and CNRS chart of ethics) and scientific misconduct and misbehaviors.

**Results:**

Our results revealed that although majority of participants declared familiar with ethical principles governing research that involves human subjects (79.5%), the overall mean score achieved on their knowledge questions was 46%. Only 27.4% are aware of the presence of the Lebanese National Consultative Committee on Ethics (LNCCE), with only half of them aware of its functions and only 25.7% know about the charter of ethics and guiding principles of scientific research in Lebanon. Significant higher levels of research ethics knowledge were recorded among Ph.D. degree-holding subjects, higher university positions as in professors, research ethics trainings-attendees, and physicians with prior research experience. A significant correlation was observed between knowledge of research ethics principles and positive attitudes toward research ethics principles. Noteworthy, we found that more than one third of participants have reported witnessing scientific misconduct and misbehaviors at some period of their careers.

**Conclusions:**

The presence of low mean awareness levels regarding research ethical principles among the study population of physicians and high levels of perception of scientific misconduct raises concern on the importance of implementing proper training for physicians on research ethics.

## Background

Challenges of the research ethical committees (REC) in developing countries receive high concerns regarding their capacity building and optimal functionality [1–6]. These challenges include lack of proper training, insufficient diversity in the members, and limited resources [2–6]. In Lebanon, although several decrees exist to regulate medical research, specific regulations regarding research ethics are still under development. Medical research ethics is mentioned in the law no. 288 [7] regulating medical practice of physicians. The *Lebanese Code of Medical Ethics* was first written in 1994 and amended lastly in 2004 when the *Rights of Patients and Informed Consent* article was introduced [8]. Unfortunately, many Lebanese hospitals do not have ethics committees and even if they do exist, they do not fulfill the role that they are intended to play [8]. In 2001, the National Lebanese Consultative Committee on Ethics (LNCCE) was established. As mentioned in their mission statement, the committee “conducts studies and provides advices, recommendations and suggestions on ethical issues related to individuals or groups and raised by research and applications in the field of life sciences and health” [9]. The last report published by the committee was in 2017, which explains its limited function [10]. Lately in 2016, the National Council for Scientific Research (CNRS) in Lebanon launched the Charter of ethics providing set of guidelines to researchers involved in research aiming to “define the basic principles of ethics and regulations that should be adopted and strictly followed by researchers and institutions in Lebanon for a responsible conduct of research in the different disciplines of science” [11].

Misconduct is defined by the U.S. Department of Health and Human Services (HHS) office of research integrity (ORI) as the “fabrication, falsification, or plagiarism in proposing, performing, or reviewing research, or in reporting research results” [12]. However, any deviations from the research ethical rules – observed as abstinence in proper consenting, undisclosed risks and misuse of physicians’ influence – are also considered as research misconduct and scientific misbehaviors [13, 14]. The true extend of research misconduct is still unclear. Many contributing factors were postulated regarding research misconduct mainly the environment surrounding the researchers, such as pressures for promotion, competition, and most essential the lack of training in research ethics [15, 16]. Thus, many studies have been conducted to assess the knowledge and the attitude of medical researchers regarding research ethical rules and guidelines [17–20].

Overall, research ethics practice showed some serious problems where in one study, more than 35% of participants found providing research details for patients un-necessary, 11.2% found fabricating data an acceptable behavior, and less than half received training in research ethics, with overall average score to assess research knowledge not exceeding 50%. In another study, 77.3% of the participants expressed concern about research misconduct occurrence and 71.8% were aware of research ethics regulations [19, 20]. Although only few physicians receive proper training in research ethics, qualified physicians should be expected to know more about research ethics guidelines in general beside their clinical skills [21].

To the best of our knowledge, only one study in the Middle East region evaluated the knowledge and the attitudes of Egyptian dental faculty regarding research ethics, so reports tackling this fundamental issue in this region are very few. This study aims at assessing the awareness, knowledge and attitude of Lebanese physicians regarding international and national research ethics guidelines and scientific misconduct and misbehaviors.

## Methods

### Study design

This cross-sectional study was conducted over a 6-months period (during February – July 2019) and was directed by the Faculty of Medicine, Beirut Arab University, Beirut, Lebanon.

The cohort in our study included Lebanese Arab physicians. No exclusion criteria were set. Email addresses of residents, faculty members and clinical affiliated doctors and professors from the seven faculties of Medicine in Lebanon (namely: Beirut Arab University, American University of Beirut, Lebanese University, University of Balamand, Lebanese American University, Holy Spirit University of Kaslik, and University Saint-Joseph of Beirut) and their affiliated hospitals were obtained. To note, the faculties of Medicine in Lebanon are not only localized in the capital, Beirut, but in different geographical areas across the country (Hadath, Byblos, El-Koura, Jounieh, and Beirut). An online questionnaire using the web link on LIME QUESTIONNAIRES was then distributed by sending emails including an online-filled questionnaire and consent form. Yet, not all the questionnaires were filled out online and some were presented in person in an interview format. Data collected using direct interviewing after participants’ consenting constituted 110 of 331 participants and was distributed among physicians across Lebanon.

### Data collection methods

The study was based on a questionnaire adopted from two previous studies [17, 22] and was divided into four parts. The first three parts were adopted and modified from a previously developed questionnaire used to assess research knowledge and attitude in Middle East region and permission was obtained from the corresponding author to use parts from the published questionnaire [17]. The fourth part tackled research misconduct and was prepared based on a previously developed questionnaire [22]. The questionnaire was adjusted to address all our study objectives (Additional file 1). Approximately, a questionnaire required 20 min to be filled.

The questionnaire was composed of four parts:

- Part 1: General information about physicians and their exposure to research ethics guidelines (10 questions)
- Part 2: Awareness and knowledge regarding research ethics principles (25 questions)
- Part 3: Attitudes towards research ethics (10 questions)
- Part 4: Perception of scientific misconduct in the workplace (18 questions) [22]

A pilot study was performed prior to commencement of the data collection to validate the modified questionnaire.

### Ethical aspects

Ethical clearance was approved by the Institutional Review Board (IRB) of Beirut Arab University prior to commencement of the study under the number (2019H-0075-M-R-0303). All data were collected anonymously. No names or other identifiers were requested from participants to be included in the questionnaire. All questionnaires were filled and collected only after participants gave their consent by clicking on the continue button (Online questionnaire) or by signing the consent form (Interview based questionnaires). The adopted consent form followed the university IRB requirement. This research study is considered to be minimal risk; the risks associated with this study are the same as what participants face every day.

To ensure accuracy, the principal investigator or study staff reviewed the collected data forms on a regular basis during the study period for data completeness as well as protocol compliance. During the recruitment phase, the rate of subject accrual and compliance with inclusion/exclusion criteria was reviewed monthly. Then it was done every 2 months to ensure that a sufficient number of participants are enrolled and that they meet eligibility criteria and the targeted ethical goals outlined in the grant proposal including providing consents to participate and publish anticipated data collected.

The principal investigator designated an independent monitor to perform an independent review of ongoing study progress and safety. The independent monitor does not have any conflicts of interest related to the study. His role was to frequently assess the study progress and safety, collect progress reports, and supervise participants’ recruitment.

### Statistical analyses

The collected data were coded, entered into the Statistical Package for the Social Sciences (SPSS) statistical software (version 23) and verified for any error in data entry. Data was normally distributed. Independent student *t-test*, ANOVA test, and Pearson correlation coefficient were used for analytical statistics and means, and frequencies were used in order to report all participants’ responses. Chi-square or Fisher’s exact test were reported for descriptive statistics. Least significance difference was used to detect statistical significance within the independent factors.

For knowledge regarding research ethics principles, physicians were asked 10 questions about research ethics guidelines and regulations. Each correct answered question was given a score of one and means were calculated. Regarding the attitudes, physicians were also asked 10 questions and answers ranged from strongly agree, agree to uncertain, disagree and strongly disagree. Categories were collapsed into strongly agree/agree on one hand, and uncertain/disagree or strongly disagree on the other hand. Positive attitudes were given a score of one and means were calculated. *p-*value of 0.05 or less was considered significant. In the scientific misconduct perception part of the questionnaire, never, seldom, occasionally, and frequently were grouped respectively. Very low was grouped with low, and high and very high were grouped accordingly.

## Results

### Physicians’ characteristics

This study included a total of 331 physicians (61.6% males and 38.4% females). The distribution of the physicians in relation with their sociodemographic factors is outlined in Table 1. The mean age of physicians was 40.66 ± 13.75. Among our study participants, 72% held only M.D. degrees, 12.1% held master’s degrees, 10.9% held Ph.D. degrees, and 5% were board certified. Categorizing physicians according to their professional status revealed that almost half of them are clinicians (51.1%), 17.3% are resident doctors, and the rest are either research officers or academic personnel (professors, lecturers, etc.). Regarding prior research exposure, almost 75% of physicians had performed or participated in research projects including humans or human subject materials. In addition, 46.3% of the physicians did not receive any training in research ethics (workshop or course). The mean years of work experience was reported to be 12.45 ± 12.90 years.

**Table 1:** General characteristics of the participants (A). Association of gender and professional status with prior research experience and prior training in research ethics (B)

**(A)**		
	**Mean ± SD**	
**Age**	40.66 ± 13.751	
	**Number (n)**	**Percentage (%)**
**Gender (323)**		
Female	124	38.4
Male	199	61.6
**Level of Education (322)**		
M.D.	232	72.0
Masters	39	12.1
Ph.D.	35	10.9
Board certified	16	5.0
**Professional status (307)**		
Professor	25	8.1
Associate Professor	22	7.2
Assistant Professor	19	6.2
Senior Lecturer	26	8.5
Clinician	157	51.1
Resident doctors	53	17.3
Research Officers	5	1.6
**Medical Speciality (331)**		
Internal Medicine	44	13.3
Obstetrics/ Gynaecology	28	8.5
Paediatric	53	16.0
Surgery	47	14.2
Others	159	48.0
	**Mean ± SD**	
**Years of experience (292)**	12.45 ± 12.90	
**Prior research involvement (331)**		
Yes	245	74
No	86	26
**Prior training in research ethics (313)**		
Yes	168	53.7
No	145	46.3
**(B)**		
	**Prior research involvement**	
	Yes (%)	No (%)
**Gender (*p*** **= 0. 896)**		
Female	73.4	26.6
Male	74.4	25.6
**Level of Education (*p*** **= 0.007)**		
M.D.	69.4	30.6
Masters	79.5	20.5
Ph.D.	91.4	8.6
Board certified	93.8	6.3
**Professional status (*p*** **= 0.000)**		
Professor	92.0	8.0
Associate Professor	95.5	4.5
Assistant Professor	100.0	0.0
Senior Lecturer	80.8	19.2
Clinician	70.1	29.9
Resident doctors	58.5	41.5
Research Officers	100.0	0.0
**Medical Speciality (*p*** **= 0.047)**		
Internal Medicine	79.5	20.5
Obstetrics/ Gynaecology	82.1	17.9
Paediatric	56.6	43.4
Surgery	78.7	21.3
Others	75.5	24.5
**Prior training in research ethics (*p*** **= 0.000)**		
Yes	60	40
No	34.6	65.4
	**Prior training in research ethics**	
	Yes (%)	No (%)
**Gender (*p*** **= 0. 55)**		
Women	51.8	48.2
Men	55.4	44.6
**Level of Education (*p*** **= 0.07)**		
M.D.	50.5	49.5
Masters	55.6	44.4
Ph.D.	74.3	25.7
Board certified	53.3	46.7
**Professional status (*p*** **= 0.003)**		
Professor	84.0	16.0
Associate Professor	47.6	52.4
Assistant Professor	68.4	31.6
Senior Lecturer	61.5	38.5
Clinician	43.1	56.9
Resident doctors	62.3	30.2
Research Officers	60.0	40.0
**Medical Speciality (*p*** **= 0.641)**		
Internal Medicine	62.8	37.2
Obstetrics/ Gynaecology	46.4	53.6
Paediatric	49.0	51.0
Surgery	55.3	44.7
Others	53.4	46.6

Associations between prior research experience and prior research ethics training with the gender, the educational level, current position, and medical speciality were performed (Table 1). Research experience was significantly higher in physicians holding a Ph.D. or board certified (*p* = 0.007), in higher academic positions (*p* = 0.000), gynaecologists (*p* = 0.03), and those with prior research ethics trainings (*p* = 0.000). However, gender was not associated with prior research experience. Regarding prior research ethics training, an association was only observed with the professional status of physicians (*p* = 0.003).

### Awareness of research ethics regulations and committees in Lebanon

Physicians’ self-reported awareness was assessed in Table 2. Almost 80% of physicians declared that they were aware about ethics principles in human subjects’ research. Significant associations were found between the awareness and professional status of physicians (*p* = 0.000), prior research experience (*p* = 0.000) and prior research ethics training (*p* = 0.000).

**Table 2:** Awareness of physicians regarding of research ethics guidelines: Association between responses and independent factors

All physicians	Yes (%)	No (%)
**Are you familiar with ethical principles that govern conducting research involving human subjects?**	248 (79.5)	64 (20.5)
**Are you familiar with ethical principles that govern conducting research involving human subjects?**		
	**Yes (%)**	**No (%)**
**Gender (*p*** **= 0. 15)**		
Female	74.8	25.2
Male	81.8	18.2
**Level of Education (*p*** **= 0.057)**		
M.D.	75.2	24.8
Masters	84.6	15.4
Ph.D.	91.2	8.8
Board certified	93.3	6.7
**Professional status (*p*** **= 0.000)**		
Professor	95.8	4.2
Associate Professor	100.0	0.0
Assistant Professor	88.9	11.1
Senior Lecturer	96.2	3.8
Clinician	74.3	25.7
Resident doctors	60.4	39.6
Research Officers	100.0	0.0
**Medical Speciality (*p*** **= 0.053)**		
Internal Medicine	77.3	22.7
Obstetrics/ Gynaecology	71.4	28.6
Paediatric	67.3	32.7
Surgery	89.4	10.6
Others	82.6	17.4
**Prior research involvement (0.000)**		
Yes	88.9	11.1
No	51.3	48.7
**Prior training in research ethics (0.000)**		
Yes	91.6	8.4
No	65.2	34.8

Although only 62.5% of the physicians knew about a research ethics committee and 68.7% acknowledged the presence of such committee at their institutions, 90% thought that its existence would be helpful. However, almost 40% were not aware of the function of these committees. Surprisingly, only 55.9% knew about the research ethics laws in Lebanon, 27.4% knew about the presence of the Lebanese National Consultative Committee on Ethics (LNCCE), and 25.7% acknowledged the charter of ethics and guiding principles of scientific research in Lebanon developed by CNRS (Table 3).

**Table 3:** Physicians’ awareness regarding research ethics committees in Lebanon

	Yes N (%)	No N (%)	Uncertain N (%)
**1. Is there a Research Ethical training program available for all Academics at your University? (285)**	145 (50.9)	97 (34)	43 (15.1)
**2. Do you know any committees/organizations that review the ethical aspects of research? (309)**	193 (62.5)	103 (33.3)	13 (4.2)
**3. Are you fully aware of the functions of ethics committees? (312)**	182 (58.3)	130 (41.7)	
**4. Does your Faculty have a research ethics committee? (310)**	213 (68.7)	34 (11)	63 (20.3)
**4. Do you think that the existence of such a research ethics committee would be helpful? (319)**	288 (90.3)	23 (7.2)	8 (2.5)
	**Yes N (%)**	**No N (%)**	**I don’t know N (%)**
**5. To your knowledge, are there laws regulating research ethics in Lebanon? (313)**	175 (55.9)	49 (15.7)	89 (28.4)
	**Yes N (%)**	**No N (%)**	
**6. Do you know the Lebanese National Consultative Committee on Ethics (LNCCE)? (317)**	87 (27.4)	230 (72.6)	
**7. If yes, are you aware about of this committee role? (86)**	51 (59.3)	35 (40.7)	
**8. Did you hear about the charter of ethics and guiding principles of scientific research in Lebanon developed by CNRS? (276)**	71 (25.7)	205 (74.3)	

### Knowledge of research ethics principle

To evaluate their knowledge, physicians were also asked questions about research ethics guidelines and related committees in Lebanon (Table 4; Additional files 2 and 3). Although majority of respondents declared being aware of such guidelines (Table 2), the calculated mean level of awareness score (based on 10 questions and a score over 10) was relatively low (4.6 ± 1.72). Physicians’ knowledge was significantly higher among Ph.D. holders (*p* = 0.01), advanced academic position personnel (*p* = 0.000), physicians with prior research experience (*p* = 0.002) and those who had prior research ethics training (*p* = 0.001). No association was observed with age and years of experience.

**Table 4:** Knowledge of physicians regarding research ethics principles and its association with independent variables

	Mean ± SD (Over 10)	
**Knowledge of physicians (331)**	4.6 ± 1.72	
	**r correlation**	***p*Value**
Age	0.6	0.92
	**Mean ± SD**	
**Gender (323)**		
Female	4.72 ± 1.64	0.413
Male	4.56 ± 1.70	
**Level of Education (322)**		
M.D.	4.57 ± 1.66	0.01
Ph.D.	5.05 ± 1.85	
Board certified	4.91 ± 1.48	
Masters	3.50 ± 1.55	
**Professional status (307)**		
Professor	5.76 ± 1.54	0.000
Associate Professor	5.32 ± 1.32	
Assistant Professor	5.21 ± 2.37	
Senior Lecturer	4.77 ± 1.63	
Clinician	4.31 ± 1.62	
Resident doctors	3.81 ± 1.06	
Research Officers	5.00 ± 1.58	
**Medical Speciality (331)**		
Internal Medicine	4.68 ± 1.61	0.8
Obstetrics/Gynaecology	4.28 ± 1.24	
Paediatric	4.47 ± 1.47	
Surgery	4.59 ± 1.59	
Others	4.67 ± 1.93	
**Prior research Involvement (331)**		
Yes	4.76 ± 1.76	
No	4.13 ± 1.51	
**Prior training in research ethics (302)**		
Yes	4.98 ± 1.8	0.001
No	4.43 ± 1.36	
	**r correlation**	
**Years of experience (272)**	6.7	0.25

### Attitudes of physicians towards practices in research ethics in Lebanon

Attitudes of physicians toward research ethics practices and affecting factors were then assessed (Table 5; Additional file 4). For this purpose, the physicians were asked 10 questions regarding research ethics practices after which “level of positive attitudes” score was determined. The mean score was 7.73 ± 2.21. The score was significantly higher in females (*p* = 0.01) and senior lecturers (p = 0.01) with no significant association with prior research experience or research ethics training. Interestingly, a significant moderate positive correlation was observed between research ethics knowledge and physicians’ attitudes toward practices of research ethics.

**Table 5:** Association between physicians’ attitudes toward research ethics practices and independent variable

	Mean ± SD	
Attitudes of physicians (10 questions) (328)	7.73 ± 2.21	
	**r correlation**	***p*Value**
**Age**	1.7	0.76
	**Mean ± SD**	
**Gender (321)**		
Female	8.14 ± 2.12	0.01
Male	7.52 ± 2.18	
**Level of Education (320)**		
MD	7.74 ± 2.25	0.996
PhD	7.74 ± 2.20	
Board certified	7.77 ± 1.94	
Masters	7.88 ± 1.96	
**Professional status (305)**		
Professor	8.08 ± 2.22	0.01
Associate Professor	8.41 ± 2.26	
Assistant Professor	8.06 ± 2.24	
Senior Lecturer	8.81 ± 1.44	
Clinician	7.35 ± 2.34	
Resident doctors	7.26 ± 1.74	
Research Officers	8.00 ± 1.22	
**Medical Speciality (328)**		
Internal Medicine	7.52 ± 2.37	0.84
Obstetrics/Gynaecology	7.82 ± 1.87	
Paediatric	7.89 ± 2.25	
Surgery	7.49 ± 1.82	
Others	7.80 ± 2.33	
**Prior research involvement (328)**		
Yes	7.86 ± 2.10	0.09
No	7.36 ± 2.47	
**Prior training in research ethics (312)**		
Yes	7.85 ± 2.13	0.708
No	7.76 ± 1.13	
	**r correlation**	
**Years of experience (270)**	6.7	0.25
**Knowledge of research ethics**	44.9	0.000

### Perception of scientific misconduct and misbehavior among physicians conducting research

Physicians were asked to rate the frequency of several acts of scientific misconduct and misbehaviors they have ever witnessed at their workplace (Table 6). As outlined, 58.5% of physicians reported witnessing scientific misconduct in terms of plagiarism at some period of their careers, 46.7% reported witnessing data falsification, and 41.9% reported witnessing data fabrication. Besides, 52% of physicians reported scientific misbehavior mainly about disagreement authorship.

**Table 6:** Perceived scientific misconduct in the workplace by physicians conducting research. *n* = 248

	Never/ Seldom Percentage (%)	Occasionally/Frequently Percentage (%)
**Plagiarism**	41.5	58.5
**Falsifying Data**	53.6	46.7
**Fabricating Data**	58.1	41.9
**Intentional protocol violation related to subject enrolment**	62.5	37.5
**Selective dropping of data from ‘Outlier’ cases**	56.9	43.1
**Falsification of Biosketch, resume, reference list**	60.9	39.1
**Disagreement about authorship**	48	52
**Pressure from study sponsor**	56.6	43.4

### Attitude of physicians toward research misconduct and misbehaviors and factors affecting their occurrence

Lastly, physicians’ attitudes regarding responsibility and factors affecting the occurrence of scientific misconduct and misbehavior were assessed (Tables 7 and 8). 50.5% of physicians disagreed that the responsibility of scientific integrity of a study lies with the principal investigator only, and 67.1% believed that dishonesty and misrepresentation (such as suppression of relevant findings, or knowingly, recklessly or by gross negligence presenting a flawed data interpretation) are uncommon in the society. When asked about the factors that might affect committing a research misconduct at their institutions, 70.7% of physicians highly and very highly agreed that those factors encompass the chances of getting caught for scientific misconduct if it occurs and 77.5% of physicians highly and very highly agreed that their own understanding of the rules and procedures related to scientific misconduct is a major factor as well. Yet, the most effective among those factors was believed to be the effectiveness of the institution’s rules and procedures for reducing scientific misconduct (78.6% of participants highly/very highly agreed).

**Table 7:** Attitudes of physicians toward research misconduct

	Agree N (%)	Disagree N (%)	I don’t know N (%)
**1. I think the responsibility for the scientific integrity of a study lies with the principal investigator only (307)**	101 (32.9)	155 (50.5)	51 (16.6)
**2. All professional education programs should include information about standards of research ethics (307)**	219 (71.3)	73 (23.8)	15 (4.9)
**3. I feel uncomfortable talking with researchers about unethical behaviour (301)**	156 (51.8)	115 (38.2)	30 (10)
**4. Dishonesty and misrepresentation of data are common in society and do not really hurt anybody (307)**	65 (21.2)	206 (67.1)	36 (11.7)

**Table 8:** Factors affecting scientific misconduct occurrence

	Very low/ Low	High/ Very High
**1. Severity of penalties for scientific misconduct (304)**	141 (46.4)	163 (53.6)
**2. Chances of getting caught for scientific misconduct if it occurs (300)**	88 (29.3)	212 (70.7)
**3. Researchers’ understanding of rules and procedures related to scientific misconduct (297)**	80 (26.9)	217 (73.1)
**4. Your own understanding of rules and procedures related to scientific misconduct. (298)**	67 (22.5)	231 (77.5)
**5. Researchers’ support of rules and procedures related to scientific misconduct. (295)**	63 (21.4)	232 (78.6)
**6. The effectiveness of your institution’s rules and procedures for reducing scientific misconduct (295)**	55(18.4)	240 (81.4)

## Discussion

Ethics has occupied a very special place in Medicine in the last 50 years. Until last few decades, researchers were believed to have strong morals and be more honest than normal citizens. However, nowadays, better knowledge of research ethics guidelines – such as safety, respect of autonomy, and social benefit of research outcome – has become a must to maintain the trust of the patient. Misconduct reporting is increasing but it is difficult yet to ascertain about the truth behind these statistics. Based on available data gathered from the U.S. government, one out of 100,000 scientists was reported to commit a fraud [23] and in a different counting one in 10,000 scientists [24]. On the other hand, paper retraction on PubMed has a 0.002% frequency. This frequency speculates that 0.02 to 0.2% of papers in the literature are fraudulent [25]. During the data auditing by the U.S. Food and Drug Administration (F.D.A.) between 1977 and 1990, 2% of clinical researchers judged guilty of serious scientific misconduct [26].

Research on human subject is continuously increasing in the Middle East and North Africa (MENA) region and especially in Lebanon with more than 200 clinical trials mostly phase III being registered in the Ministry of Public Health since 2014 [27]. Hence, current data are scarce, and there is a great need to gather these data to determine how knowledgeable clinical researchers are and evaluate research integrity and responsible conduct crucial for producing reliable evidence-based research. In line with this, Fanelli’s systematic review and meta-analysis study addressing research ethics and misconduct did not include any study from the MENA region; yet, many cases have occurred, reflecting the dire need to conduct such study in Lebanon [28]. Our results presented herein shed light on research ethics trainings in Lebanon regarding the international guidelines and the national laws and decrees and the new CRNS chart of ethics.

This study aimed to fill the gap in the knowledge about the attitude, knowledge, awareness, and perception of Lebanese physicians regarding research ethics guidelines and rules. Results of this study reveal that majority of physicians are aware of research ethics guidelines, although their calculated mean level of awareness is low. This low level of awareness was surprisingly associated with higher education levels among PhD degree-holding subjects, higher positions, those who attend research ethics training, and those who have prior research experience. Besides, despite the foundation of a national committee on ethics (LNCCE)) in 2001, only 27.4% of our study physicians knew about it. Also, only 25.7% acknowledged the presence of a charter of ethics and guiding principles of scientific research in Lebanon that was developed by the CNRS in 2016.

In the Middle East region, only one study evaluated the knowledge and attitudes of Egyptian faculties regarding research ethics. Similar to our findings, authors showed that the mean score of physicians’ knowledge was 42% - which is very close to our mean (46%) – and that the awareness toward research ethics guidelines is associated with professional status and prior research ethics training. However, in contrast to our results, prior research experience was not associated with awareness [17]. In other studies, 77.3% of the participants expressed concern about research misconduct occurrence and 71.8% of participants were aware of research ethics regulations [19, 20]. Although only few physicians receive proper training in research ethics, qualified physicians should be expected to know about research ethics guidelines in general beside their clinical skills [21].

In our study, results showed that despite the low mean difference, physicians attending research ethics workshops or courses scored statistically higher. This result is in accordance with a Nigerian study showing that 3-days workshop in research ethics have improved both physicians’ knowledge and research ethics principles application [29]. Similar conclusions were also observed in other studies [30–32]. Yet, another Nigerian study demonstrated that attendance of research ethics training or seminar did not significantly increase the knowledge of participants [19]. None of these studies have identified the type and lengths effect of the research trainings which might explain the observed discrepancy between studies.

In this study, physicians were asked whether they have ever witnessed any scientific misconduct at their workplace. Noteworthy, large number of physicians reported witnessing scientific misconduct, mainly disagreement about authorship, plagiarisms, and fabricated and falsified data. In a similar Norwegian study, 27% of physicians declared that they are aware of research misconduct in their institutions; however, 42% acknowledged that this information was not publicly known [33]. Moreover, higher prevalence of misconduct was observed in an international questionnaire of biostatisticians in ‘51%’, and in questionnaire of British medical consultants (56%) [15, 16]. In a meta-analysis of questionnaires involving self-reported and/or witnessed scientific misconduct, 14.12% of physicians admitted witnessing falsification and up to 72% witnessed other questionable research practices [28]. Another study showed high percentage of physicians who witnessed one or more form of scientific misconduct in their workplace [22].

Nowadays, societies are continuously losing trust because of research misconduct and the lack of accountability in biomedical researchers [34]. They are concerned about the respectful of patient rights, the dignity and autonomy of individuals, and the integrity of the research outcomes. Therefore, ethical rules and public trust in medical research are inseparable. Any research misconduct would jeopardize the public faith in medical research and its crucial role in the advancement of scientific knowledge. This public concern may be overcome by enhancing the knowledge of researchers in the ethics guidelines.

Lastly, we acknowledge that this study might have some limitations. This is a cross-sectional study based on a relatively small sample collected by convenience and questions that might not cover the wide range of research ethics principles but represented the basic information that physicians must require prior to research involving human subjects.

## Conclusions

In conclusion, our study demonstrated that Lebanese physicians have low knowledge regarding research ethics guidelines and regulations; however, they have positive attitudes towards practices in research ethics. Moreover, research ethics training positively impact the development of physicians’ knowledge and attitudes toward research ethics, where majority declared being aware of research ethics guidelines which reflects increased knowledge. Besides, a significant moderate positive correlation was found between research ethics regulations knowledge and physicians’ attitudes toward practices of research ethics, signifying positive attitudes. Significant positive associations were found between the awareness and professional status of physicians, prior research experience, and prior research ethics training. A significant moderate positive correlation was also demonstrated between research ethics knowledge and physicians’ attitudes toward practices of research ethics. Results showed the high rates of research misconduct and scientific misbehavior perceived by our participants where almost half of physicians reported witnessing scientific misconduct at some period of their careers. Our study also shed the light on the importance of reinforcing the function and the role of research ethics committees in establishing institutional and national educational programs in research ethics, in accordance with international effort to enhance research ethics principles knowledge between researchers.

## Supplementary information

**Additional file 1.** Questionnaire form.

**Additional file 2: Supplementary Table I.** Least significant difference between physicians’ knowledge across their level of education.

**Additional file 3: Supplementary Table II.** Least significant difference between physicians’ knowledge across their current position.

**Additional file 4: Supplementary Table III.** Least significant difference between physicians’ attitudes across their current position.

**Additional file 5.** Raw data as spss file.

## Data Availability

The datasets supporting the conclusions of this article are included within the article (Additional file 5).

## Abbreviations

LNCCE: Lebanese National Consultative Committee on Ethics
REC: Research Ethical Committees
CNRS: National Council for Scientific Research
ORI: Office of Research Integrity
HHS: U.S. Department of Health and Human Services
IRB: Institutional Review Board ()
SPSS: Statistical Package for the Social Sciences statistical software
F.D.A.: U.S. Food and Drug Administration
MENA: Middle East and North Africa
