# Supplementary material for: Knowledge and attitudes of physicians toward research ethics and scientific misconduct in Lebanon
Source: BMC Med Ethics. 2020 May 14;21:39. doi: 10.1186/s12910-020-00475-5 (PMC7227247; doi:10.1186/s12910-020-00475-5)
Supplement: Supplementary file 1 — Additional file 1. Questionnaire form. [file 12910_2020_475_MOESM1_ESM.doc]

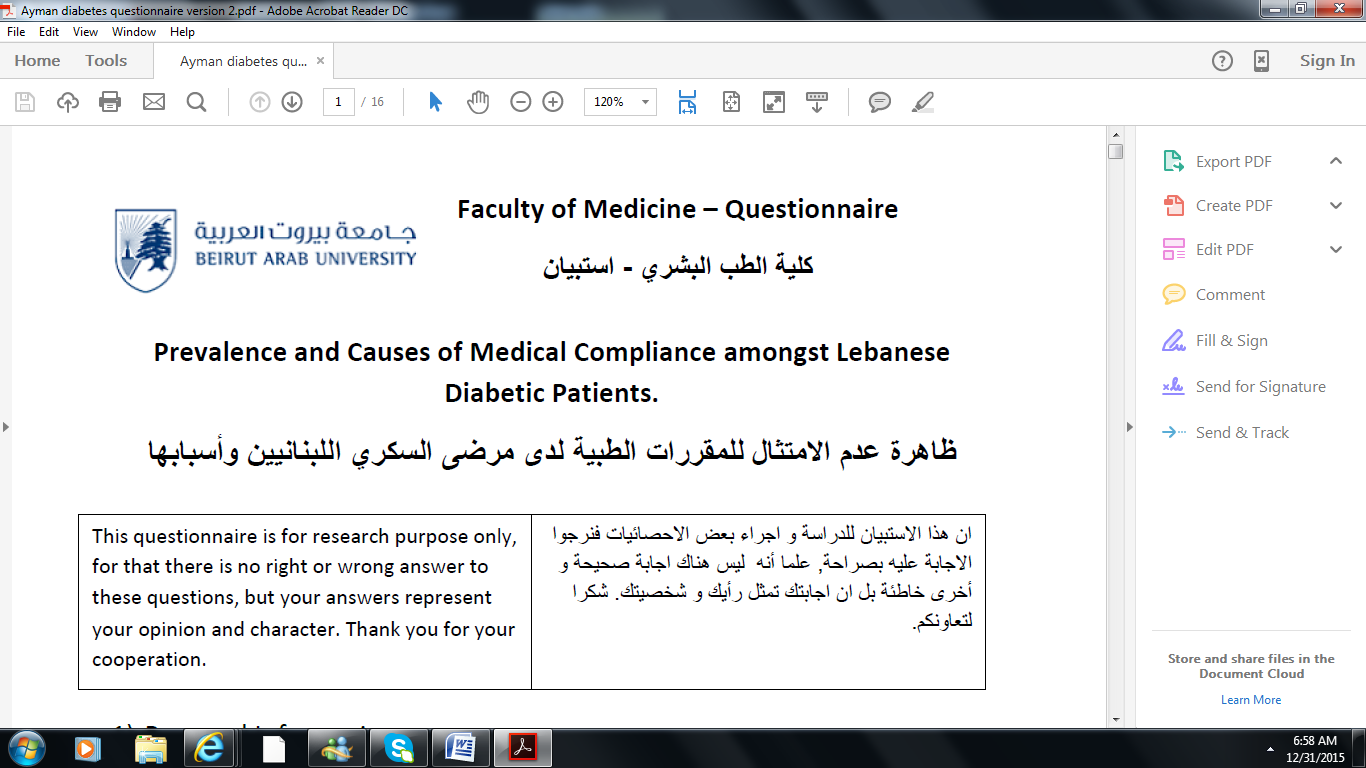
**Knowledge and attitudes of clinical researchers regarding research ethics and misconduct in Lebanon**

**PART I: Personal Background information**

**1. Age: ……………………………**

**2. Gender**

□ Male □ Female

**3.** **Highest level of education**

□ MD Year……….. University………….

□ Masters Year……….. University………….

□ PhD Year……….. University………….

□ Boarded certified Year……….. Bard………….

**4. Current Position :**

□ Professor

□ Associate Professor

□ Assistant Professor

□ Senior Lecturer/ Lecturer

**5. Professional status**

□ Consultants/clinicians

□ Resident doctors

□ Research officers

**6. Specialty**

□ Internal Medicine

□ Gynecology

□ Pediatric

□ Surgery

□ Family Medicine

□Other, Specify:

# **7. The number of years of experience in your current work:**

# **……………………………..**

**8. Publications including master and doctor thesis:**

- Did they include human subjects?

□ Yes □No How many…….

- Did they include human biological samples (e.g., blood , tissue, urine, teeth, saliva.., etc)? □ Yes □No How many………

**9. Number of thesis you supervised: ………..**

- Did they include human subjects?

□ Yes □ No

- Did they include human biological samples (e.g. blood , tissue, urine, teeth, saliva.., etc) ? □ Yes □ No

**10. Number of research projects you contributed in …………**

- Did they include human subjects?

□ Yes □ No

- Did they include human biological samples (e.g. blood, tissue, urine, teeth, saliva..etc.) ?

□ Yes □ No

**PART II: Awareness about research ethics**

This section asks about your awareness with Research ethics and Ethics committees. Please check one of the following options:

|  | **Yes** | **No** | **Uncertain** |
| --- | --- | --- | --- |
| 1. Are you familiar with ethical principles that govern conducting research involving human subjects? | □ | □ | □ |
| 2) Are you a member of a research ethics committee? | □ | □ | □ |
| 3.1) Did you attend a course on Research ethics or Bioethics? | □ | □ | □ |
| 3.2) Did you attend workshops on Research Bioethics? | □ | □ | □ |
| 4) Is there a Research Ethical training program available for all Academics at your University? | □ | □ | □ |
| 5) Do you know any committees/organizations that review the ethical aspects of research? | □ | □ | □ |
| 6) Do you think that the existence of such a research ethics committee would be helpful? | □ | □ | □ |
| 7) Are you fully aware of the functions of ethics committees? | □ | □ | □ |
| 8) Does your Faculty have a research ethics committee? | □ | □ | □ |

**Please check one of the following answers:**

**9- What is the importance of ethics?**

□ To regulate the lives of people

□ To guide the life of people

□ To protect the welfare of people

□ All of the above

□ None of the above/please write your opinion …………….……………

**10- Which of the following is considered guidelines in research ethics?**

□ Nuremberg code

□ Declaration of Helsinki

□ Belmont Report

□ Council of the International Organizations of the Medical Sciences (CIOMS)

□ All of the above.

**11- If a research ethics committee exists, what do you think is its role?**

□ review the ethical aspects of the research

□ determine whether informed consent is needed

□ review the scientific design of the research

□protect the welfare and rights of the subjects in the research

□make research more difficult to perform

□other , specify:

**12- Do you think the patient should be informed about the full details of the research, including all risks and benefits?**

□ Yes

□ No

□ I don't know

**13- If you think informed consent should be obtained, should the patient always have to sign a written form?**

□ Yes

□ No

□ I don't know

**14- Regarding vulnerable groups such as children (ages less than 18) and the mentally ill, do you think they should give an informed consent?**

□ Yes

□ No

□ I don't know

**15- If no, from whom will you get the informed consent on their behalf?**

**……………………………………………………………………………………………………………………………………………………………………………………**

**16- If no one is available to give informed consent for a vulnerable person, is it proper to enroll these individuals in research?**

□ Yes

□ No

□ I don't know

**17- Regarding the use of Human biological tissue samples (solid tissues, blood, other body fluids)** **do you think patients should provide their informed consent?**

□ Yes

□ No

□ I don't know

**18- Regarding the Human biological tissue samples taken in hospitals, university hospitals and institutions, who do you think owns this material after it is collected from the patient?**

a. The hospital or institute

b. The patient

c. The investigator

**19- Do you agree in using experimental animals in bio-medical research?**

□ Yes

□ No

□ I don't know

**20- If yes, do you think there are ethical rules regulating the use of experimental animals in bio-medical research?**

□ Yes

□ No

□ I don't know

**21- To your knowledge, are there laws regulating research ethics in Lebanon**?

□ Yes

□ No

□ I don't know

**22- Do you know The Lebanese National Consultative Committee on Ethics (LNCCE)?**

□ Yes

□ No

**23- If yes, are you aware about the role of this Committee?**

□ Yes

□ No

**24- Did you hear about the Charter of ethics and guiding principles of scientific research in Lebanon developed by CNRS?**

□ Yes

□ No

**25. What does you regulate research in Lebanon?**

□ Laws

□ Decree

□ Memorandum of understanding

□ Nothing

# **PART III: Attitudes towards Research Ethics**

This section asks for your opinion regarding research ethics. Please indicate the degree to which you agree or disagree with the following statements by checking one of the following options:

**(SA) (A) (D) (SD) (U)**

|  | **Strongly Agree** | **Agree** | **Disagree** | **Strongly Disagree** | **Uncertain** |
| --- | --- | --- | --- | --- | --- |
| 1. Research ethics should be taught as a mandatory module at postgraduate studies. | □ | □ | □ | □ | □ |
| 1. All investigators should have some training in research ethics. | □ | □ | □ | □ | □ |
| 1. There is a need for more emphasis on research ethics in conducting research involving human subjects. | □ | □ | □ | □ | □ |
|  | **Strongly Agree** | **Agree** | **Disagree** | **Strongly Disagree** | **Uncertain** |
| 1. When involving our patients in research that presents as more than minimal risk, we must seek an informed consent from each patient. | □ | □ | □ | □ | □ |
| 1. When obtaining data from patients, there should be measures to protect such data from accidental disclosure | □ | □ | □ | □ | □ |
| 1. Patients do not have the ability to understand research, therefore, no need to bother them with details and it is enough to get their permission to participate in a study. | □ | □ | □ | □ | □ |
| 1. If a blood sample is being obtained for clinical Laboratory tests and an investigator would like to use some of this blood for a research study, it is not necessary to obtain informed consent from the patient regarding the research study. | □ | □ | □ | □ | □ |
| 1. When conducting clinical research, patients should not be told about potential risks otherwise they may not accept to participate in the study | □ | □ | □ | □ | □ |
| 1. It is acceptable sometimes to fabricate some of the data or results to improve the outcome of the research as long as there is no harm to patients. | □ | □ | □ | □ | □ |
| 1. It is difficult to get a study published if the researcher does not follow the ethical guidelines. | □ | □ | □ | □ | □ |

**PART V: Perception of scientific misconduct in the workplace**

How much you witness the following:

|  | **Never** | **Seldom** | **Occasionally** | **Frequently** |
| --- | --- | --- | --- | --- |
| 1- Plagiarism | □ | □ | □ | □ |
| 2.1- Falsifying data (changing or omission of research results) | □ | □ | □ | □ |
| 2.2- Fabricating data (making up data) | □ | □ | □ | □ |
| 3- Intentional protocol violations related to subject enrolment | □ | □ | □ | □ |
|  | **Never** | **Seldom** | **Occasionally** | **Frequently** |
| 5- Selective dropping of data from ‘outlier’ cases | □ | □ | □ | □ |
| 6- Falsification of biosketch, resume, reference list | □ | □ | □ | □ |
| 7- Disagreements about authorship | □ | □ | □ | □ |
| 8- Pressure from study sponsor (e.g. pharmaceutical company or device company) to engage in unethical practices | □ | □ | □ | □ |

**What do you think about the following situations?**

|  | **Agree** | **Disagree** | **I Don’t know** |
| --- | --- | --- | --- |
| 9- I think the responsibility for the scientific integrity of a study lies with the principal investigator only | □ | □ | □ |
| 10- All professional education programs should include information about standards of research ethics | □ | □ | □ |
| 11- I feel uncomfortable talking with researchers about unethical behavior | □ | □ | □ |
| 12- Dishonesty and misrepresentation of data are common in society and do not really hurt any body | □ | □ | □ |

**To your opinion, how much these factors would affect scientific integrity?**

|  | **Very low** | **Low** | **High** | **Very high** |
| --- | --- | --- | --- | --- |
| 13- Severity of penalties for scientific misconduct | □ | □ | □ | □ |
| 14- Chances of getting caught for scientific misconduct if it occurs | □ | □ | □ | □ |
| 15- Researchers’ understanding of rules and procedures related to scientific misconduct | □ | □ | □ | □ |
| 16- Your own understanding of rules and procedures related to scientific misconduct. | □ | □ | □ | □ |
| 17- Researchers’ support of rules and procedures related to scientific misconduct | □ | □ | □ | □ |
| 18- The effectiveness of your institution’s rules and procedures for reducing scientific misconduct | □ | □ | □ | □ |

Thank you for your cooperation
