# Supplementary material for: Knowledge and attitudes of physicians toward research ethics and scientific misconduct in Lebanon
Source: BMC Med Ethics. 2020 May 14;21:39. doi: 10.1186/s12910-020-00475-5 (PMC7227247; doi:10.1186/s12910-020-00475-5)
Supplement: Supplementary file 2 — Additional file 2: Supplementary Table I. Least significant difference between physicians’ knowledge across their level of education. [file 12910_2020_475_MOESM2_ESM.docx]

**Additional file 2. Supplementary Table I.** Least significant difference between physicians’ knowledge across their Level of Education.

| What is your highest level of education? | What is your highest level of education? | Mean Difference | *p* value |
| --- | --- | --- | --- |
|  |  |  |  |
| Masters | MD | -1.06897 | .013 |
|  | PhD | -1.55128 | .002 |
|  | Board certified | -1.41429 | .005 |
