# Supplementary material for: Knowledge and attitudes of physicians toward research ethics and scientific misconduct in Lebanon
Source: BMC Med Ethics. 2020 May 14;21:39. doi: 10.1186/s12910-020-00475-5 (PMC7227247; doi:10.1186/s12910-020-00475-5)
Supplement: Supplementary file 3 — Additional file 3: Supplementary Table II. Least significant difference between physicians’ knowledge across their current position. [file 12910_2020_475_MOESM3_ESM.docx]

**Additional file 3. Supplementary Table II.** Least significant difference between physicians’ knowledge across their current position.

| What is your current position? | What is your current position? | Mean Difference | *p* value |
| --- | --- | --- | --- |
| Clinician | Professor  Associate Professor  Assistant Professor  Resident doctors | -1.45427  -1.01245  -.90479  .49441 | .000  .005  .018  .049 |
| Resident doctors | Professor  Associate Professor  Assistant Professor  Senior Lecturer  Clinician | -1.94868  -1.50686  -1.39921  -.95791  -.49441 | .000  .000  .001  .011  .049 |
