# Supplementary material for: Knowledge and attitudes of physicians toward research ethics and scientific misconduct in Lebanon
Source: BMC Med Ethics. 2020 May 14;21:39. doi: 10.1186/s12910-020-00475-5 (PMC7227247; doi:10.1186/s12910-020-00475-5)
Supplement: Supplementary file 4 — Additional file 4: Supplementary Table III. Least significant difference between physicians’ attitudes across their current position. [file 12910_2020_475_MOESM4_ESM.docx]

**Additional file 4. Supplementary Table III.** Least significant difference between physicians’ attitudes across their current position.

| What is your current position? | What is your current position? | Mean Difference | *p* value |
| --- | --- | --- | --- |
| Clinician | Associate Professor  Senior Lecturer | -1.06294  -1.46154 | .031  .001 |
| Resident doctors | Associate Professor  Senior Lecturer | -1.14494  -1.54354 | .037  .003 |
